# 2-Propynyl 2-hydroxy­benzoate

**DOI:** 10.1107/S160053680905421X

**Published:** 2009-12-24

**Authors:** Stephan M. Levonis, Milton J. Kiefel, Todd A. Houston, Peter C. Healy

**Affiliations:** a Institute for Glycomics, Griffith University, Gold Coast Campus, Gold Coast 4222, Australia; b Eskitis Institute for Cell and Molecular Therapies, Griffith University, Nathan Campus, Brisbane 4111, Australia

## Abstract

The title compound, C_10_H_8_O_3_, has been synthesized as part of our investigations into the generation of new anti­bacterial agents and serves as a building block for the synthesis of compound libraries. The compound crystallizes with two independent mol­ecules in the asymmetric unit. The *transoid* propynyl ester groups are coplanar with the 2-hydroxy­benzoate group with maximum deviations of −0.3507 (3) and 0.1591 (3) Å for the terminal carbons, with intra­molecular O—H⋯O hydrogen bonding providing rigidity to the structure and ensuring that the reactivity of the alkyne is not compromised by steric factors. The propynyl group forms inter­molecular C—H⋯O inter­actions with the phenolic O atom. Supra­molecular chains along the *b* axis are found for both mol­ecules with links by weak O—H⋯O inter­molecular inter­actions in the first independent mol­ecule and C—H⋯O inter­actions in the second.

## Related literature

For background to Cu(I)-mediated azide–alkyne cyclo­additions, see: Houston *et al.* (2008 ▶); Wilkinson *et al.* (2009 ▶). For the biological use of salicylates, see: Sox & Olson (1989 ▶). For background to boric acid-mediated esterification, see: Houston *et al.* (2004 ▶, 2007 ▶); Levonis *et al.* (2007 ▶). For stereochemistry, see: Wilkinson *et al.* (2006 ▶); Wiberg & Laidig (1987 ▶). For previous synthesis of the title compound and its anti-tumour activity, see: Jung *et al.* (1997 ▶).
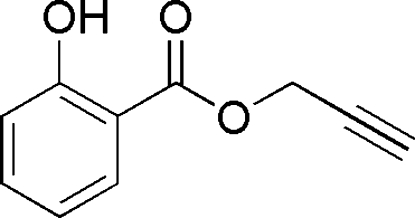


## Experimental

### 

#### Crystal data

C_10_H_8_O_3_
*M* *_r_* = 176.16
Monoclinic, 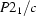

*a* = 18.7150 (14) Å
*b* = 12.7972 (10) Å
*c* = 7.2310 (7) Å
β = 90.191 (8)°
*V* = 1731.8 (3) Å^3^
*Z* = 8
Mo *K*α radiation
μ = 0.10 mm^−1^
*T* = 296 K
0.36 × 0.30 × 0.12 mm

#### Data collection

Oxford-Diffraction GEMINI S Ultra diffractometer
Absorption correction: multi-scan (*CrysAlis RED*; Oxford Diffraction, 2009 ▶) *T*_min_ = 0.965, *T*_max_ = 0.988
10756 measured reflections
3081 independent reflections
1941 reflections with *I* > 2σ(*I*)
*R*_int_ = 0.031

#### Refinement

*R*[*F* ^2^ > 2σ(*F* ^2^)] = 0.039
*wR*(*F* ^2^) = 0.098
*S* = 0.93
3081 reflections
235 parameters
H-atom parameters constrained
Δρ_max_ = 0.14 e Å^−3^
Δρ_min_ = −0.14 e Å^−3^

### 

Data collection: *CrysAlis CCD* (Oxford Diffraction, 2009 ▶); cell refinement: *CrysAlis RED* (Oxford Diffraction, 2009 ▶); data reduction: *CrysAlis RED*; program(s) used to solve structure: *SIR97* (Altomare *et al.*, 1999 ▶); program(s) used to refine structure: *SHELXL97* (Sheldrick, 2008 ▶); molecular graphics: *ORTEP-3 for Windows* (Farrugia, 1997 ▶); software used to prepare material for publication: *PLATON* (Spek, 2009 ▶) and *publCIF* (Westrip, 2010 ▶).

## Supplementary Material

Crystal structure: contains datablocks global, I. DOI: 10.1107/S160053680905421X/tk2601sup1.cif

Structure factors: contains datablocks I. DOI: 10.1107/S160053680905421X/tk2601Isup2.hkl

Additional supplementary materials:  crystallographic information; 3D view; checkCIF report

## Figures and Tables

**Table 1:** Hydrogen-bond geometry (Å, °)

*D*—H⋯*A*	*D*—H	H⋯*A*	*D*⋯*A*	*D*—H⋯*A*
O1—H1⋯O7	0.90	1.86	2.6193 (16)	141
O1—H1⋯O7^i^	0.90	2.55	3.2081 (17)	130
O11—H11⋯O17	0.90	1.82	2.6007 (18)	144
C10—H10⋯O11^ii^	0.95	2.38	3.310 (2)	165
C16—H16⋯O17^iii^	0.96	2.48	3.291 (2)	143
C20—H20⋯O1^iv^	0.95	2.46	3.340 (2)	154

Symmetry codes: (i) 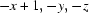; (ii) 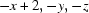; (iii) 
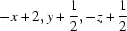
; (iv) 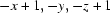.

**Fig. 1.**
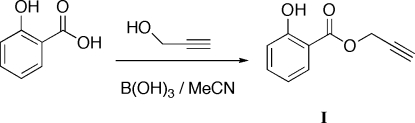
Reaction scheme for the preparation of the title compound (I).

**Fig. 2.**
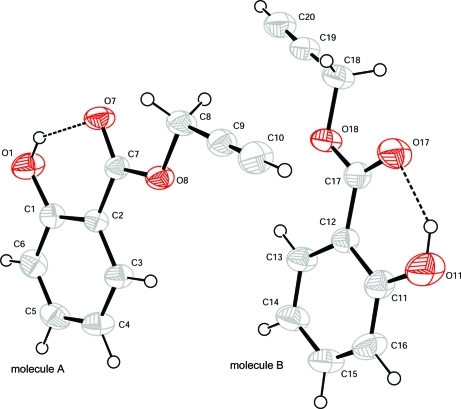
View of the two independent molecules in (I) with the atom numbering scheme. Displacement ellipsoids for non-H atoms are drawn at the 40% probability level.

**Fig. 3.**
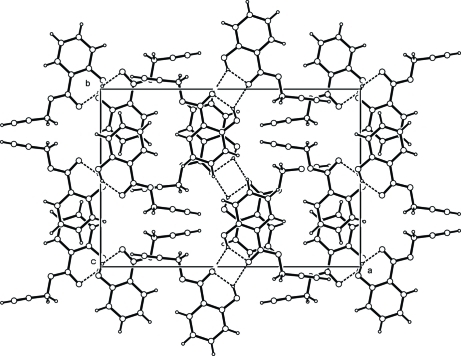
Crystal packing in the structure of (I), viewed down the c axis.

## supplementary crystallographic information

### Comment

In an attempt to identify new antibacterial compounds, we have assembled a diverse range of azide and alkyne coupling partners for the purpose of creating compound libraries using Cu(I)-mediated azide-alkyne cycloadditions [CuAAC] (Houston *et al.*, 2008; Wilkinson *et al.*, 2009). Salicylates such as bismuth subsalicylate have been used for many years to treat diarrhea and other gastrointestinal disorders (Sox & Olson, 1989). We required a core salicylate scaffold that could be readily transformed into a variety of derivatives. Here, we describe the synthesis and X-ray crystal structure of 2'-propynyl 2-hydroxybenzoate (propargyl salicylate) (I) using our chemoselective method of boric acid-mediated esterification (Houston *et al.*, 2004; 2007). Borate can activate hydroxycarboxylic acids such as salicylate toward esterification under mild conditions that are tolerant to acid-labile functional groups such as alkynes. This ester was previously synthesized by alkylation for the synthesis of cobalt carbonyl complexes and study of their anti-tumour activity (Jung *et al.*, 1997).

Compound (I) was synthesized cleanly from salicylic acid and propargyl alcohol in 55% yield using 10 mol% boric acid in acetonitrile (Levonis *et al.*, 2007) (Fig. 1), and crystallizes from toluene with two independent molecules in the asymmetric unit (Fig. 2). The ester group adopts the *transoid* arrangement (Wilkinson *et al.*, 2006) as stereoelectronic requirements are met when the carbonyl bifurcates the methylene H atoms (Wiberg & Laidig, 1987). This allows both p →π and n →σ* overlap from the propargylated oxygen to the carbonyl. The propynyl groups are co-planar with the 2-hydroxybenzoate; with the intra-molecular O—H···O hydrogen bond between the phenolic proton and the carbonyl oxygen providing rigidity to the structure (Table 1). These factors result in the extension of the propynyl group away from the aromatic core and ensures that the reactivity of the alkyne when using the CuAAC method is not compromised by steric constraints. In the crystal lattice, the propynyl groups form inter-molecular C—H···O interactions with the phenolic oxygen (Table 1). Supramolecular chains along the direction of the *b* axis are found for both molecules with links by weak O1—H1···O7 (molecule A) and C16—H16···O17 (molecule B) inter-molecular interactions (Table 1, Fig. 3).

### Experimental

To a stirred solution of salicylic acid (208 mg, 1.5 mmol) and propargyl alcohol (84 mg, 174 mL,3.0 mmol) in acetonitrile (3 ml) was added boric acid (9 mg, 0.15 mmol). The solution was heated and maintained at reflux for 16 h before concentrating *in vacuo*. Flash column chromatography was performed on silica using ethyl acetate as the mobile phase to yield 145 mg(55%) of (I) as a white solid. This was initially recrystallized from MeOH to furnish white needles (31%) for NMR analysis. A second recrystallization from toluene at 0°C produced single crystals suitable for X-ray diffraction analysis.

^1^H NMR (CDCl_3_ 300 MHz, 298 K) δ p.p.m. 2.53 (t, *J* = 2.4 Hz, 1H), 4.91 (s, 2H), 6.87 (ddd, *J* = 8.1, 7.35, 1.2 Hz, 1H), 6.96 (dd, *J* = 8.4, 0.9 Hz, 1H), 7.45 (ddd, *J* = 8.4, 7.2, 1.8 Hz, 1H), 7.85 (dd, *J* = 7.9, 1.8 Hz, 1H), 10.5 (s, 1H). ^13^C{^1^H} NMR (CD_3_OD, 75 MHz, 298 K) δ p.p.m. 53.7, 77.1, 78.3, 113.2, 118.5, 120.4, 130.9, 137.0, 162.9 170.5. MS(ESI–) 175.1 [M—H^+^]

### Refinement

H atoms were positioned geometrically, with C–H = 0.95 - 0.96 Å and O—H = 0.90 Å, and refined as riding on their parent atoms with *U*_iso_(H) = 1.2*U*_eq_.

### Crystal data

**Table:**

C_10_H_8_O_3_	*F*(000) = 736
*M**_r_* = 176.16	*D*_x_ = 1.351 Mg m^−^^3^
Monoclinic, *P*2_1_/*c*	Mo *K*α radiation, λ = 0.71070 Å
Hall symbol: -P 2ybc	Cell parameters from 3378 reflections
*a* = 18.7150 (14) Å	θ = 3.2–25.0°
*b* = 12.7972 (10) Å	µ = 0.10 mm^−^^1^
*c* = 7.2310 (7) Å	*T* = 296 K
β = 90.191 (8)°	Block, colourless
*V* = 1731.8 (3) Å^3^	0.36 × 0.30 × 0.12 mm
*Z* = 8	

### Data collection

**Table:**

Oxford-Diffraction GEMINI S Ultra diffractometer	3081 independent reflections
Radiation source: Enhance (Mo) X-ray Source	1941 reflections with *I* > 2σ(*I*)
graphite	*R*_int_ = 0.031
Detector resolution: 16.0774 pixels mm^-1^	θ_max_ = 25.2°, θ_min_ = 3.2°
ω and φ scans	*h* = −22→22
Absorption correction: multi-scan (*CrysAlis RED*; Oxford Diffraction, 2009)	*k* = −15→15
*T*_min_ = 0.965, *T*_max_ = 0.988	*l* = −8→8
10756 measured reflections	

### Refinement

**Table:**

Refinement on *F*^2^	Primary atom site location: structure-invariant direct methods
Least-squares matrix: full	Secondary atom site location: difference Fourier map
*R*[*F*^2^ > 2σ(*F*^2^)] = 0.039	Hydrogen site location: inferred from neighbouring sites
*wR*(*F*^2^) = 0.098	H-atom parameters constrained
*S* = 0.93	*w* = 1/[σ^2^(*F*_o_^2^) + (0.0531*P*)^2^] where *P* = (*F*_o_^2^ + 2*F*_c_^2^)/3
3081 reflections	(Δ/σ)_max_ = 0.001
235 parameters	Δρ_max_ = 0.14 e Å^−^^3^
0 restraints	Δρ_min_ = −0.14 e Å^−^^3^

### Special details

**Table:**

Geometry. Bond distances, angles *etc*. have been calculated using the rounded fractional coordinates. All su's are estimated from the variances of the (full) variance-covariance matrix. The cell e.s.d.'s are taken into account in the estimation of distances, angles and torsion angles
Refinement. Refinement on *F*^2^ for ALL reflections except those flagged by the user for potential systematic errors. Weighted *R*-factors *wR* and all goodnesses of fit *S* are based on *F*^2^, conventional *R*-factors *R* are based on *F*, with *F* set to zero for negative *F*^2^. The observed criterion of *F*^2^ > σ(*F*^2^) is used only for calculating -*R*-factor-obs *etc*. and is not relevant to the choice of reflections for refinement. *R*-factors based on *F*^2^ are statistically about twice as large as those based on *F*, and *R*-factors based on ALL data will be even larger.

### Fractional atomic coordinates and isotropic or equivalent isotropic displacement parameters (Å^2^)

**Table:**

	*x*	*y*	*z*	*U*_iso_*/*U*_eq_	
O1	0.47564 (6)	0.17697 (9)	0.12869 (19)	0.0639 (5)	
O7	0.57032 (6)	0.02715 (9)	0.10015 (17)	0.0548 (5)	
O8	0.68501 (5)	0.06996 (8)	0.06527 (16)	0.0471 (4)	
C1	0.53250 (9)	0.24189 (13)	0.1244 (2)	0.0435 (6)	
C2	0.60284 (8)	0.20595 (12)	0.1059 (2)	0.0370 (5)	
C3	0.65779 (8)	0.27917 (13)	0.1010 (2)	0.0464 (6)	
C4	0.64437 (10)	0.38407 (14)	0.1112 (3)	0.0554 (7)	
C5	0.57450 (11)	0.41802 (14)	0.1295 (3)	0.0580 (7)	
C6	0.51933 (9)	0.34830 (14)	0.1376 (2)	0.0535 (7)	
C7	0.61613 (8)	0.09414 (12)	0.0911 (2)	0.0387 (6)	
C8	0.69957 (9)	−0.04016 (13)	0.0400 (3)	0.0529 (7)	
C9	0.77295 (9)	−0.05066 (13)	−0.0228 (2)	0.0497 (7)	
C10	0.83132 (10)	−0.06146 (15)	−0.0747 (3)	0.0633 (8)	
O11	1.00806 (6)	0.04958 (11)	0.2637 (2)	0.0843 (6)	
O17	0.91622 (6)	−0.09764 (10)	0.33340 (19)	0.0675 (5)	
O18	0.80791 (6)	−0.05357 (8)	0.43315 (15)	0.0496 (4)	
C11	0.95424 (9)	0.11680 (14)	0.3039 (3)	0.0528 (7)	
C12	0.88641 (8)	0.08199 (13)	0.3537 (2)	0.0428 (6)	
C13	0.83356 (9)	0.15589 (13)	0.3864 (2)	0.0516 (7)	
C14	0.84724 (10)	0.26037 (15)	0.3751 (3)	0.0624 (8)	
C15	0.91532 (11)	0.29250 (15)	0.3294 (3)	0.0661 (8)	
C16	0.96819 (10)	0.22262 (16)	0.2939 (3)	0.0641 (8)	
C17	0.87317 (9)	−0.02986 (13)	0.3700 (2)	0.0453 (6)	
C18	0.79398 (10)	−0.16364 (13)	0.4549 (3)	0.0561 (7)	
C19	0.72292 (10)	−0.17626 (13)	0.5328 (2)	0.0513 (7)	
C20	0.66642 (10)	−0.19093 (15)	0.5941 (3)	0.0627 (8)	
H1	0.48950	0.11110	0.10160	0.0760*	
H3	0.70590	0.25560	0.08950	0.0550*	
H4	0.68250	0.43350	0.10570	0.0670*	
H5	0.56540	0.49150	0.13700	0.0690*	
H6	0.47180	0.37330	0.15270	0.0630*	
H8A	0.69370	−0.07620	0.15400	0.0630*	
H8B	0.66820	−0.06890	−0.04980	0.0630*	
H10	0.87890	−0.07030	−0.11700	0.0750*	
H11	0.99390	−0.01650	0.28820	0.0990*	
H13	0.78680	0.13280	0.42100	0.0610*	
H14	0.81070	0.31060	0.39670	0.0750*	
H15	0.92580	0.36530	0.32450	0.0790*	
H16	1.01500	0.24600	0.26110	0.0790*	
H18A	0.79640	−0.19700	0.33770	0.0640*	
H18B	0.82850	−0.19340	0.53560	0.0640*	
H20	0.62010	−0.20280	0.64430	0.0760*	

### Atomic displacement parameters (Å^2^)

**Table:**

	*U*^11^	*U*^22^	*U*^33^	*U*^12^	*U*^13^	*U*^23^
O1	0.0381 (7)	0.0519 (8)	0.1017 (11)	−0.0002 (6)	0.0130 (6)	−0.0085 (7)
O7	0.0394 (7)	0.0389 (7)	0.0862 (10)	−0.0057 (6)	0.0079 (6)	0.0033 (6)
O8	0.0360 (6)	0.0325 (7)	0.0728 (8)	0.0015 (5)	0.0047 (6)	−0.0034 (5)
C1	0.0414 (10)	0.0436 (11)	0.0456 (11)	0.0020 (8)	0.0048 (8)	−0.0021 (8)
C2	0.0388 (9)	0.0340 (9)	0.0381 (10)	0.0011 (7)	0.0050 (7)	0.0004 (7)
C3	0.0418 (10)	0.0402 (11)	0.0574 (12)	−0.0010 (8)	0.0100 (8)	−0.0030 (8)
C4	0.0619 (12)	0.0377 (11)	0.0668 (14)	−0.0063 (9)	0.0118 (10)	−0.0065 (9)
C5	0.0769 (14)	0.0356 (11)	0.0614 (13)	0.0098 (10)	0.0049 (10)	−0.0041 (9)
C6	0.0504 (11)	0.0489 (12)	0.0612 (13)	0.0144 (9)	0.0068 (9)	−0.0059 (9)
C7	0.0343 (9)	0.0388 (10)	0.0431 (10)	−0.0016 (8)	0.0025 (7)	0.0002 (8)
C8	0.0461 (10)	0.0331 (10)	0.0794 (14)	0.0048 (8)	0.0039 (9)	−0.0018 (9)
C9	0.0470 (11)	0.0380 (11)	0.0641 (13)	0.0043 (8)	0.0025 (9)	−0.0048 (9)
C10	0.0509 (12)	0.0542 (13)	0.0847 (15)	0.0051 (10)	0.0100 (11)	−0.0090 (10)
O11	0.0449 (8)	0.0664 (10)	0.1419 (14)	0.0016 (7)	0.0332 (8)	−0.0043 (9)
O17	0.0504 (8)	0.0455 (8)	0.1068 (12)	0.0114 (6)	0.0168 (7)	−0.0037 (7)
O18	0.0498 (7)	0.0343 (7)	0.0649 (8)	−0.0014 (5)	0.0163 (6)	0.0002 (5)
C11	0.0421 (10)	0.0540 (12)	0.0624 (13)	−0.0011 (9)	0.0113 (9)	−0.0022 (9)
C12	0.0413 (9)	0.0418 (10)	0.0454 (11)	−0.0025 (8)	0.0078 (8)	−0.0011 (8)
C13	0.0455 (10)	0.0427 (11)	0.0667 (13)	−0.0010 (8)	0.0172 (9)	0.0000 (8)
C14	0.0636 (13)	0.0421 (12)	0.0817 (15)	0.0012 (10)	0.0155 (11)	0.0021 (10)
C15	0.0756 (14)	0.0418 (11)	0.0810 (15)	−0.0126 (11)	0.0095 (12)	0.0039 (10)
C16	0.0517 (12)	0.0622 (14)	0.0785 (15)	−0.0170 (10)	0.0158 (10)	0.0031 (11)
C17	0.0406 (10)	0.0439 (11)	0.0513 (12)	0.0025 (8)	0.0059 (8)	−0.0013 (8)
C18	0.0615 (12)	0.0350 (11)	0.0718 (13)	0.0001 (9)	0.0112 (10)	0.0010 (9)
C19	0.0570 (12)	0.0366 (11)	0.0602 (12)	−0.0055 (9)	0.0079 (9)	0.0017 (8)
C20	0.0575 (12)	0.0481 (12)	0.0826 (15)	−0.0045 (10)	0.0124 (11)	0.0012 (10)

### Geometric parameters (Å, °)

**Table:**

O1—C1	1.351 (2)	C5—H5	0.9600
O7—C7	1.2143 (19)	C6—H6	0.9500
O8—C7	1.3394 (18)	C8—H8B	0.9500
O8—C8	1.447 (2)	C8—H8A	0.9500
O1—H1	0.9000	C10—H10	0.9500
O11—C11	1.357 (2)	C11—C12	1.394 (2)
O17—C17	1.214 (2)	C11—C16	1.381 (3)
O18—C17	1.340 (2)	C12—C17	1.458 (2)
O18—C18	1.441 (2)	C12—C13	1.389 (2)
O11—H11	0.9000	C13—C14	1.364 (3)
C1—C6	1.387 (2)	C14—C15	1.380 (3)
C1—C2	1.401 (2)	C15—C16	1.359 (3)
C2—C3	1.392 (2)	C18—C19	1.455 (3)
C2—C7	1.456 (2)	C19—C20	1.163 (3)
C3—C4	1.368 (2)	C13—H13	0.9600
C4—C5	1.385 (3)	C14—H14	0.9500
C5—C6	1.366 (3)	C15—H15	0.9500
C8—C9	1.454 (2)	C16—H16	0.9600
C9—C10	1.165 (3)	C18—H18A	0.9500
C3—H3	0.9500	C18—H18B	0.9500
C4—H4	0.9500	C20—H20	0.9500
			
O1···O7	2.6193 (16)	C20···O1^ii^	3.340 (2)
O1···O7^i^	3.2081 (17)	C20···C8^xi^	3.519 (3)
O1···C20^ii^	3.340 (2)	C3···H13^iv^	2.9700
O7···O1^i^	3.2081 (17)	C4···H13^iv^	3.0100
O7···O1	2.6193 (16)	C7···H1	2.3800
O7···C6^iii^	3.416 (2)	C13···H3^vii^	3.0300
O7···O7^i^	3.0795 (17)	C14···H3^vii^	3.0800
O8···C4^iv^	3.419 (2)	C15···H11^x^	3.1000
O11···O17	2.6007 (18)	C17···H11	2.3400
O11···C10^v^	3.310 (2)	C18···H8A	3.0800
O17···O11	2.6007 (18)	C19···H18A^xi^	3.0600
O17···C16^vi^	3.291 (2)	C19···H8A	3.0700
O17···C10	3.379 (3)	C20···H8B^xii^	3.0100
O18···C9	3.3593 (18)	C20···H8A^xi^	3.0500
O1···H20^ii^	2.4600	H1···O7	1.8600
O7···H6^iii^	2.7800	H1···C7	2.3800
O7···H8B	2.4600	H1···O7^i^	2.5500
O7···H1	1.8600	H3···C13^iv^	3.0300
O7···H8A	2.6900	H3···C14^iv^	3.0800
O7···H1^i^	2.5500	H3···H13^iv^	2.4100
O8···H3	2.4100	H3···H14^iv^	2.5500
O11···H15^vi^	2.7400	H3···O8	2.4100
O11···H10^v^	2.3800	H4···H13^iv^	2.5200
O17···H11	1.8200	H6···O7^viii^	2.7800
O17···H18A	2.5800	H8A···C18	3.0800
O17···H18B	2.5200	H8A···C19	3.0700
O17···H16^vi^	2.4800	H8A···C20^ix^	3.0500
O18···H13	2.4200	H8A···O7	2.6900
C4···O8^vii^	3.419 (2)	H8B···C20^xiii^	3.0100
C4···C7^vii^	3.523 (3)	H8B···O7	2.4600
C5···C7^vii^	3.429 (3)	H10···O11^v^	2.3800
C6···O7^viii^	3.416 (2)	H11···O17	1.8200
C7···C4^iv^	3.523 (3)	H11···C17	2.3400
C7···C5^iv^	3.429 (3)	H11···C15^vi^	3.1000
C8···C20^ix^	3.519 (3)	H11···H15^vi^	2.2800
C9···C17	3.409 (2)	H13···O18	2.4200
C9···O18	3.3593 (18)	H13···C3^vii^	2.9700
C10···O11^v^	3.310 (2)	H13···C4^vii^	3.0100
C10···C18^ix^	3.593 (3)	H13···H3^vii^	2.4100
C10···C17	3.332 (3)	H13···H4^vii^	2.5200
C10···O17	3.379 (3)	H14···H3^vii^	2.5500
C14···C15^vii^	3.584 (3)	H15···O11^x^	2.7400
C15···C14^iv^	3.584 (3)	H15···H11^x^	2.2800
C15···C16^vii^	3.504 (3)	H16···O17^x^	2.4800
C16···O17^x^	3.291 (2)	H18A···O17	2.5800
C16···C15^iv^	3.504 (3)	H18A···C19^ix^	3.0600
C17···C9	3.409 (2)	H18B···O17	2.5200
C17···C10	3.332 (3)	H20···O1^ii^	2.4600
C18···C10^xi^	3.593 (3)		
			
C7—O8—C8	115.11 (12)	O8—C8—H8A	110.00
C1—O1—H1	110.00	C9—C10—H10	180.00
C17—O18—C18	115.08 (13)	O11—C11—C16	118.02 (16)
C11—O11—H11	109.00	C12—C11—C16	119.96 (16)
O1—C1—C6	117.52 (15)	O11—C11—C12	122.01 (16)
O1—C1—C2	122.74 (15)	C11—C12—C13	118.42 (15)
C2—C1—C6	119.73 (15)	C11—C12—C17	119.34 (15)
C1—C2—C3	118.42 (14)	C13—C12—C17	122.24 (14)
C1—C2—C7	119.36 (14)	C12—C13—C14	121.55 (16)
C3—C2—C7	122.22 (14)	C13—C14—C15	118.69 (17)
C2—C3—C4	121.57 (15)	C14—C15—C16	121.51 (18)
C3—C4—C5	119.13 (16)	C11—C16—C15	119.84 (18)
C4—C5—C6	120.89 (17)	O17—C17—O18	121.26 (15)
C1—C6—C5	120.25 (16)	O17—C17—C12	124.83 (15)
O7—C7—O8	121.63 (14)	O18—C17—C12	113.91 (14)
O7—C7—C2	124.67 (14)	O18—C18—C19	108.46 (14)
O8—C7—C2	113.70 (13)	C18—C19—C20	177.08 (19)
O8—C8—C9	107.93 (13)	C12—C13—H13	119.00
C8—C9—C10	178.35 (19)	C14—C13—H13	119.00
C2—C3—H3	119.00	C13—C14—H14	121.00
C4—C3—H3	119.00	C15—C14—H14	120.00
C5—C4—H4	120.00	C14—C15—H15	119.00
C3—C4—H4	121.00	C16—C15—H15	119.00
C4—C5—H5	119.00	C11—C16—H16	120.00
C6—C5—H5	120.00	C15—C16—H16	121.00
C1—C6—H6	120.00	O18—C18—H18A	109.00
C5—C6—H6	119.00	O18—C18—H18B	110.00
O8—C8—H8B	110.00	C19—C18—H18A	110.00
C9—C8—H8A	110.00	C19—C18—H18B	110.00
H8A—C8—H8B	109.00	H18A—C18—H18B	109.00
C9—C8—H8B	109.00	C19—C20—H20	180.00
			
C8—O8—C7—O7	3.0 (2)	C2—C3—C4—C5	0.9 (3)
C8—O8—C7—C2	−176.81 (14)	C3—C4—C5—C6	0.1 (3)
C7—O8—C8—C9	168.65 (13)	C4—C5—C6—C1	−1.1 (3)
C18—O18—C17—O17	0.6 (2)	O11—C11—C12—C13	−177.77 (16)
C18—O18—C17—C12	−178.56 (14)	O11—C11—C12—C17	2.3 (3)
C17—O18—C18—C19	177.13 (13)	C16—C11—C12—C13	2.1 (3)
C6—C1—C2—C7	−179.66 (13)	C16—C11—C12—C17	−177.86 (17)
O1—C1—C6—C5	−178.39 (16)	O11—C11—C16—C15	178.65 (19)
C2—C1—C6—C5	1.1 (2)	C12—C11—C16—C15	−1.2 (3)
O1—C1—C2—C7	−0.2 (2)	C11—C12—C13—C14	−1.6 (2)
C6—C1—C2—C3	−0.1 (2)	C17—C12—C13—C14	178.33 (16)
O1—C1—C2—C3	179.35 (14)	C11—C12—C17—O17	−4.3 (2)
C1—C2—C3—C4	−0.9 (2)	C11—C12—C17—O18	174.83 (15)
C7—C2—C3—C4	178.62 (16)	C13—C12—C17—O17	175.82 (15)
C3—C2—C7—O8	−2.5 (2)	C13—C12—C17—O18	−5.1 (2)
C1—C2—C7—O7	−2.8 (2)	C12—C13—C14—C15	0.2 (3)
C1—C2—C7—O8	177.01 (13)	C13—C14—C15—C16	0.7 (3)
C3—C2—C7—O7	177.69 (15)	C14—C15—C16—C11	−0.2 (3)

Symmetry codes: (i) −*x*+1, −*y*, −*z*; (ii) −*x*+1, −*y*, −*z*+1; (iii) −*x*+1, *y*−1/2, −*z*+1/2; (iv) *x*, −*y*+1/2, *z*−1/2; (v) −*x*+2, −*y*, −*z*; (vi) −*x*+2, *y*−1/2, −*z*+1/2; (vii) *x*, −*y*+1/2, *z*+1/2; (viii) −*x*+1, *y*+1/2, −*z*+1/2; (ix) *x*, −*y*−1/2, *z*−1/2; (x) −*x*+2, *y*+1/2, −*z*+1/2; (xi) *x*, −*y*−1/2, *z*+1/2; (xii) *x*, *y*, *z*+1; (xiii) *x*, *y*, *z*−1.

### Hydrogen-bond geometry (Å, °)

**Table:**

*D*—H···*A*	*D*—H	H···*A*	*D*···*A*	*D*—H···*A*
O1—H1···O7	0.90	1.86	2.6193 (16)	141
O1—H1···O7^i^	0.90	2.55	3.2081 (17)	130
O11—H11···O17	0.90	1.82	2.6007 (18)	144
C10—H10···O11^v^	0.95	2.38	3.310 (2)	165
C16—H16···O17^x^	0.960	2.48	3.291 (2)	143
C20—H20···O1^ii^	0.95	2.46	3.340 (2)	154

Symmetry codes: (i) −*x*+1, −*y*, −*z*; (v) −*x*+2, −*y*, −*z*; (x) −*x*+2, *y*+1/2, −*z*+1/2; (ii) −*x*+1, −*y*, −*z*+1.
